# Bovine herpesvirus 4 based vector as a potential oncolytic-virus for treatment of glioma

**DOI:** 10.1186/1743-422X-7-298

**Published:** 2010-11-03

**Authors:** Marco Redaelli, Carla Mucignat-Caretta, Andrea Cavaggioni, Antonio Caretta, Domenico D'Avella, Luca Denaro, Sandro Cavirani, Gaetano Donofrio

**Affiliations:** 1 Department of Human Anatomy and Physiology, University of Padova, Italy; 2 Department of Neuroscience, University of Padova, Italy; 3 Department of Pharmaceutical Sciences, University of Parma, Italy; 4 Department of Animal Health, University of Parma, Italy

## Abstract

The application of gene therapy for malignant gliomas is still under study and the use of specific vectors represents an important contribution. Here, we investigated bovine herpesvirus 4 (BoHV-4), which is non-pathogenic if injected into the rodent brain. We show that the vector can infect mouse, rat and human glioma cell lines and primary cultures obtained from human glioblastoma in vitro. BoHV-4 was injected into a tumour grown in rat brain. Although virus expression was scattered across the tumour mass, it was mainly located in the peripheral area of larger gliomas. These data support BoHV-4 as a candidate vector for glioma treatment.

## Findings

Gene therapy for the selective treatment of brain tumours is intriguing, particularly given the limited efficacy of currently available therapeutic options. In thirteen studies that performed clinical trials with gene therapy, results showed an increase in mean survival time ranging from 8.9 months to 14.4 months [1]. The optimization of potential vectors is essential for clinical effectiveness of cancer gene therapy.

Bovine herpesvirus 4 (BoHV-4) belongs to the Herpesviridae family, gamma-herpesviridae subfamily [2]. The monocyte/macrophage lineage is one of the sites of persistence of infection in cattle, a natural host, and in experimental hosts the rabbit [3]. BoHV-4 is able to replicate in a broad range of host species both *in vivo *and in *vitro *[4]. BoHV-4 replicates and causes a cytopathic effect (CPE) in a large number of immortalized cell lines and primary cultures [3,5,6].

Although BoHV-4 is not considered a neurotropic virus, it has been isolated in peripheral and central nervous systems during persistent infection [7]. While BoHV-4 induces apoptosis in some cancer cell lines [6], the association between the virus and disease is at present unclear. BoHV-4 does not replicate in mouse or rat brain, but reporter gene expression has been shown in ependymal cells and the rostral migratory stream (RMS) area after the injection into the lateral ventricle of both mouse and rat brain [8]. These data prompted us to investigate the use of BoHV-4 as a vector for gene therapy or oncolytic therapy of brain tumours.

As a first approach, the replicating competence of BoHV-4 was initially tested in vitro using three different cell lines, the GL261 mouse glioblastoma cell line, the F98 rat glioma cell line and the GLI36 human glioma cell line. Cells were maintained in monolayer using complete growth medium (CGM) with 90% Dulbecco Modified Eagle's Medium (DMEM), 10% FBS, 100 I.U./ml penicillin, 10 μg/ml streptomycin, 10 μg/ml tetracycline, 25 μg/ml Plasmocin (InVivogen, Milan, Italy). Cells were incubated at 37°C in a humidified environment with 95% air and 5% CO_2_, for up to 80-90% confluence (4-6 days).

**Figure 1:**
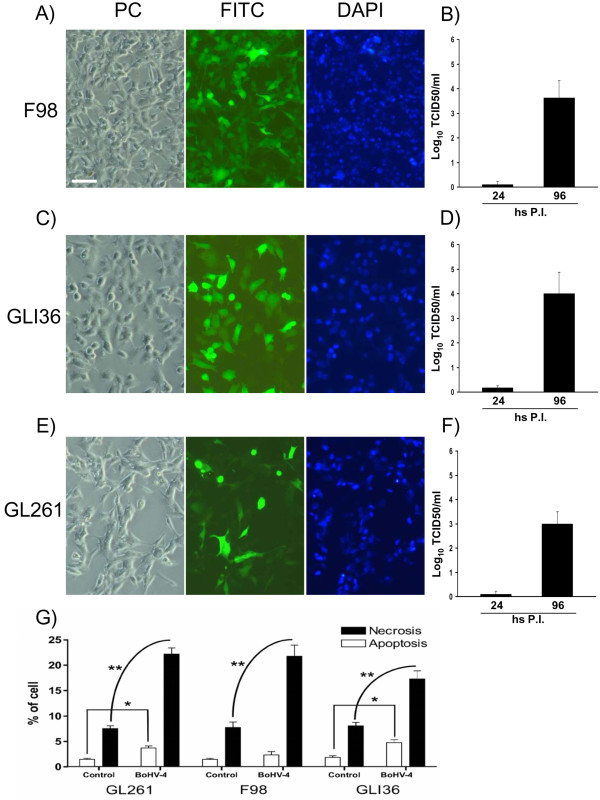
**Representative pictures (10×) of BoHV-4-EFGPΔTK infected F98 (A), GLI36 (C) and GL261 (E) cells at 96 hours (hs) post infection (P.I.), visualized by phase contrast (PC) fluorescence with a FITC filter for EGFP expression or with DAPI filter for nuclear counterstaining (bar = 100 μm)**. The respective titers (expressed as log_10 _of Tissue Cells Infectious Dose/50 [TCID_50_] per ml^-1^) of viral particles released during the time at 24 and 96 hours (hs) post infection (P.I.) are shown in B, D and F. Values are the mean ± standard error of three independent experiments. (G) GL261 mouse glioblastoma cell line (a, bar = 25 μm), F98 rat glioma cell line (b, bar = 25 μm) and GLI36 human glioma cell line (c, bar = 10 μm) infected with BoHV-4EGFPΔTK for 72 hours. CPE induced by infection shows a prevalence of necrosis (ANOVA, ** p < 0.001, *p < 0.05).

**Figure 2:**
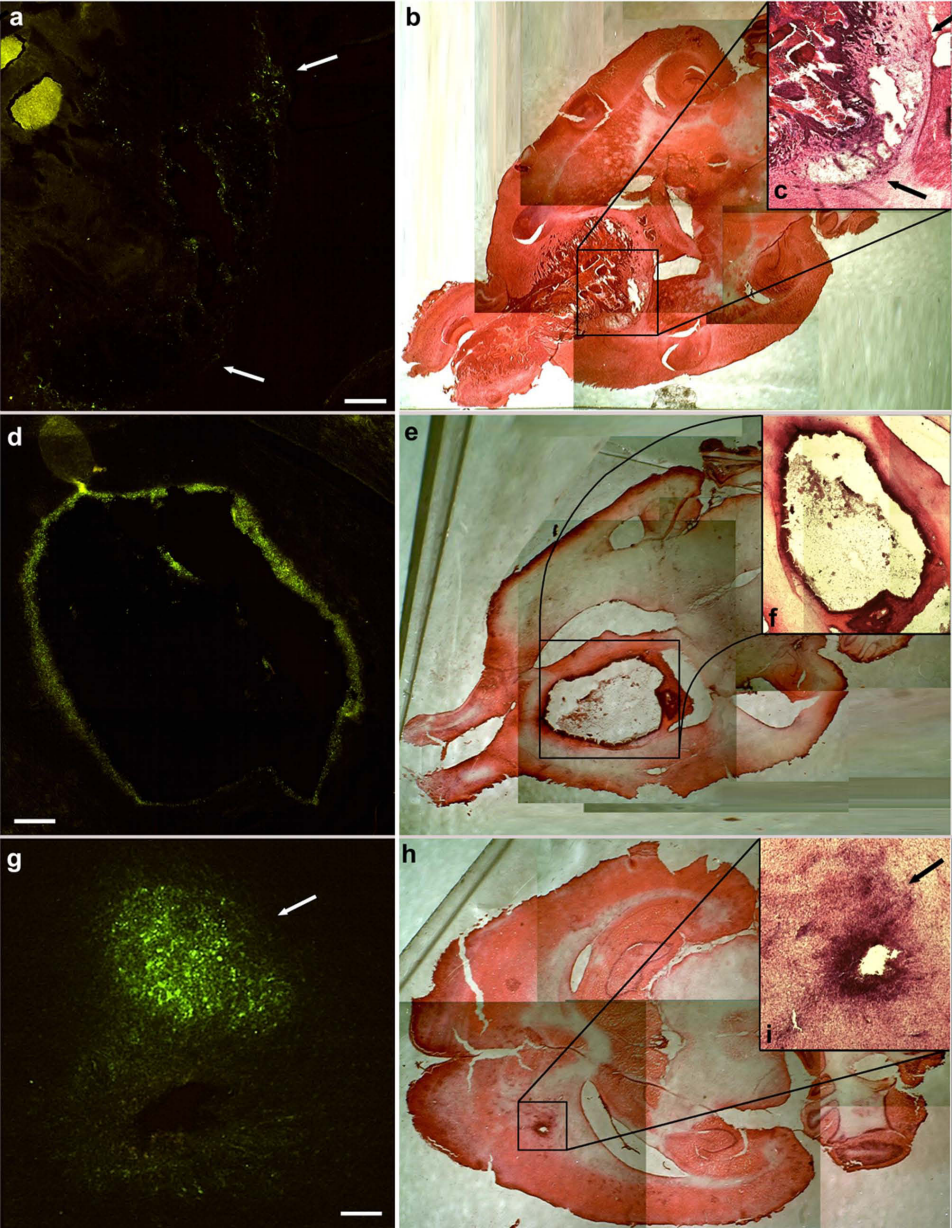
**Frozen sections (horizontal) of the BoHV-4 injected rat brain gliomas**. EGFP expression in the peripheral area of tumour 48 hours post BoHV-4 injection (a, bar = 500 μm), hematoxylin eosin of the whole section (b) with magnification of the tumour area in the insert (c). EGFP expression in the solid peripheral area of a cystic tumour 96 hours post BoHV-4 injection (d, bar = 500 μm), hematoxylin eosin of the whole section (e) with magnification of the tumour area in the insert (f). EGFP expression in the whole mass of non necrotic tumours 132 hours post BoHV-4 injection (g, bar = 150 μm), hematoxylin eosin of the whole section (h) with magnification of the tumour area in the insert (i).

**Figure 3:**
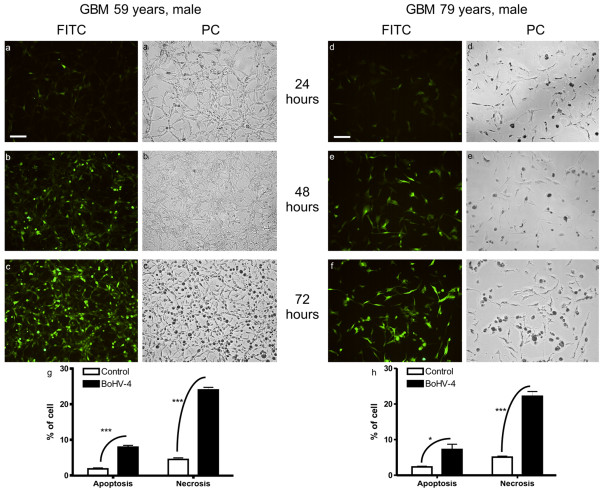
**Primary cultures from two human glioblastoma analyzed 24, 48 and 72 hours post BoHV-4EGFPΔTK infection**. The cells were visualized with a FITC filter for EGFP expression (a, b, c, d, e, f, bar = 50 μm) and by phase contrast (PC) (a_i_, b_i_, c_i_, d_i_, e_i_, f_i_). After 72 hours post infection the cultures were completely infected. CPE induced by infection shows a prevalence of necrosis (g, h, t-test, *** p < 0.001, *p < 0.05).

Infection was performed with 1 TCID50/cell of a recombinant BoHV-4 expressing EGFP (BoHV-4EGFPΔTK) [3] and its effects were observed after 24, 48, 72, 96, 144, 216 hours post infection with an epi-fluorescence microscope (Leica). Indeed BoHV-4EGFPΔTK infected, replicated and induced cytopathic effects (CPE) in all three cell lines tested (Figure 1A, C and 1E). To quantify the newly produced progeny virus, the non-penetrated infectious viral particles were inactivated by low-pH treatment after infection. Cultures were washed with medium and cultured until CPE appeared, after which 1 ml of the medium was removed from each well and centrifuged for 5 min at 3000 rpm in a bench top centrifuge to remove any cellular debris and TCID50 were determined (tittering was repeated three times for each cell line). All three cell lines sustained productive infection (Figure 1B, D and 1F). In order to analyze the CPE induced by BoHV-4EGFPΔTK, cells were fixed with methanol and stained with Wright's stain. A total of 600 cells were counted from each slide, and the percentage of apoptotic and necrotic cells was calculated. At least 6 control and 6 treated slides were counted for each treatment. Monovariate ANOVA was used to test differences in the percentage of dead cells between control and infected cells. The CPE induced in vitro by BoHV-4EGFPΔTK infection was prevalently necrosis (Figure 1G) rather than apoptosis. Similar results were obtained with Annexin V and Propidium Iodide staining (data not shown). These results, together with the data previously obtained *in vivo *where BoHV-4 did not replicate in the mouse and rat brain, but reporter gene expression was shown following injection into the mouse and rat lateral ventricle, prompted us to investigate the use of BoHV-4 as a vector for the gene therapy or as an oncolytic virus of brain tumours. Thus, a rat glioma model was constructed. Fifteen four-month-old, male Fisher rats were pre-anesthetized with isoflurane and subsequently anesthetized with zolazepam tiletamine (20 mg/kg body weight) and xylazine (75 mg/kg body weight). Eight × 10^6 ^F98 glioma cells were suspended in 8 μl DMEM and injected 1 mm anterior and 1.5 mm lateral to the bregma, 3.7 mm below the pial surface. Injection was carried out for 16 minutes and was performed using a Hamilton syringe. Animals were monitored daily for neurological signs and weight loss. At the appearance of neurological signs, animals were re-anesthetized as above and 6 μl of 10^6 ^pfu of BoHV-4EGFPΔTK were injected into the same position as the previous injection. Animals were then monitored every 12 hours. Any animals showing severe worsening of neurological conditions were humanely euthanize. Rat brains were analyzed at different post-injection times: 48, 72, 86, 96, 120, 132, 144 and 216 hours. Briefly, anesthetized rats were first perfused with PBS for 15 min and then with 4% formalin in PBS for 30 min. Brains were carefully removed, post-fixed for 2 hours in 4% formalin in PBS, equilibrated for 24 h in 30% sucrose in PBS at 4°C and frozen at -80°C until sectioning with a cryostat at 16 μm. Sections of the BoHV-4-injected, rat brain gliomas showed EGFP expression in the peripheral area of larger tumours (Figure 2a, b), scattered across the mass of smaller tumours (Figure 2c), and in the solid peripheral area of cystic tumours (Figure 2d). These same sections, following observation of EGFP expression, were then stained with hematoxylin-eosin. In order to confirm co-localization of the tumour area with EGFP-positive transduced cells, five four-month-old male rats were inoculated with 8 × 10^6 ^F98 glioma cells labelled with the red fluorescent cell linker PHK26, according to manufacturer's instructions (Sigma). Cells maintain fluorescence for more than 100 mitotic divisions [9]. When BoHV-4EGFPΔTK was injected into the rat brains at the same position as the marked glioma cells, co-localization between the red fluorescent-marked tumour area and the EGFP positive cells was observed, without detection of the EGFP signal within the brain parenchyma (data not shown). In another experiment, primary cultures from biopsies of 2 patients with glioblastoma (both males, 59 and 79 years of age respectively) were prepared. Specimens were dissociated not more than 30 minutes after surgery by shaking for 5 minutes in 0.25% Trypsin, 0.02% EDTA (1 ml/mm^3 ^tissue). The suspension was inactivated with CGM, and centrifuged at 37°C for 10 minutes at 1350 rpm. The supernatant was discharged and the pellet resuspended in 10 ml of CGM, changed every 72 hour for three weeks. Cells were then infected with BoHV-4EGFPΔTK and analyzed 24, 48 and 72 hours post infection as described above. Indeed, these primary cultures of human glioblastoma were susceptible to BoHV-4 infection as shown by EGFP expression, and also in this case infection leaded to a mainly necrotic CPE (Figure 3).

We here report the capacity of BoHV-4 to infect and replicate in glioma cell lines and glioblastoma primary cultures *in vitro *and the ability of BoHV-4 to selectively infect gliomas induced in the rat brain *in vivo*.

BoHV-4 is not oncogenic, unlike other γ-herpesviruses like KSHV, EBV and HVS [10]. In addition, the attenuation by gene inactivation is not mandatory, due to the mild pathogenicity of the virus in natural and experimental hosts. Interestingly, previous studies demonstrated that BoHV-4EGFPΔTK infection is not permissive in the rat and mouse brain [8], unlike the replication-competent behaviour of BoHV-4EGFPΔTK in a different number of cell lines *in vitro*.

The data from clinical trials underline the need to refine gene therapy protocols through combination with other therapeutic strategies or by improving the efficiency and selectivity of vectors [1]. A recent clinical trial with combined cytokine/suicide gene therapy for glioma supported the efficacy of the transduction of therapeutic genes to the targeted tumour cells in human patients [11]. These data suggest a possible application in the long-term control of tumour growth.

The present study demonstrates the safety of the vector *in vivo *and the efficiency of the transduction of the reporter gene both *in vitro *and *in vivo*. *In vitro*, the ability of BoHV-4 to infect different glioma cell lines, as demonstrated by the expression of the reporter gene, suggests the suitability of this vector for gene therapy. The selectivity of the virus for glioma cells in the nervous system and its safety have been also tested *in vivo*. The evolution of infection and the distribution of EGFP-positive cells within the tumour area shows the selectivity of the virus for the tumour cells and its oncolytic properties. Moreover the non-replicative behaviour of the virus in the brain parenchyma [8] is important for its safe use. These data are supported by the analysis of the infection in the F98-PHK26red model *in vivo*, that also confirm that BoHV-4 infection is confined to the tumour area. Lastly, the infection of human primary culture of brain tumour extends our results in rat gliomas to human gliomas.

In conclusion, the capability to establish an infection of glioma cells *in vitro*, of both immortalized cell lines as well as primary cultures, the *in vivo *non-pathogenicity and the affinity for the glioma cells *in vivo *set BoHV-4 up as a candidate for gene delivery and oncolyses to the glial tumours of the nervous system.

## List of abbreviations

BoHV-4: Bovine herpesvirus 4; CPE: Cytopathic effect; CGM: Complete growth medium; RMS: Rostral migratory stream; DMEM: Dulbecco Modified Eagle's Medium; EGFP: Enhanced green fluorescent protein; FBS: Fetal bovine serum

## Competing interests

The authors declare that they have no competing interests.

## Authors' contributions

RM: performed the experiments and wrote the paper. M-CC, CA and CA: intellectually contributed. DD and DL: provided human glioma samples. GD: Conceive the experiments, performed the experiments and wrote the paper.
